# Wisconsin Society of Veterinary Graduates

**Published:** 1895-09

**Authors:** 


### WISCONSIN SOCIETY OF VETERINARY GRADUATES.

The meeting of this Society that was announced for August 14th, at Chippewa Falls, was countermanded, and no meeting will be held until the annual meeting in February, 1896. The general lack of professional prosperity and the expense of members, incidental to the meeting, are given as influencing causes for this decision.

